# Genome Sequence of a Cluster DN1 Gordonia terrae Phage, Periwinkle

**DOI:** 10.1128/mra.00696-22

**Published:** 2022-08-25

**Authors:** Emma M. Boudreaux, Sophie B. Childs, Abigail Dichiara, Amy Hardy, Sam Kovacs, Alison F. Kueck, Parker M. Landesbergen, Melody Neely, Sally Molloy

**Affiliations:** a Molecular and Biomedical Sciences, University of Maine, Orono, Maine, USA; b The Honors College, University of Maine, Orono, Maine, USA; Queens College CUNY

## Abstract

Periwinkle is a temperate bacteriophage that was isolated on the host Gordonia terrae 3612. The genome has a length of 55,657 bp and a GC content of 62.9% and contains 109 protein-coding genes and no tRNA genes. An 8-kb region after the structural protein genes encodes eight membrane proteins, a tyrosine integrase, and an immunity repressor.

## ANNOUNCEMENT

Actinobacteriophages are extremely abundant and diverse viruses that infect bacteria within the phylum *Actinobacteria* (1–4). By studying bacteriophages through programs such as the Science Education Alliance-Phage Hunters Advancing Genomics and Evolutionary Science (SEA-PHAGES) Program, we advance our understanding of phage and bacterial diversity, evolution, and virus-host interactions (3–6). Periwinkle was isolated from a composted manure sample collected in Orono, Maine (44.915628N, 68.69072W), using Gordonia terrae 3612 (7). Soil extracts were prepared in peptone-yeast extract-calcium (PYCa) medium, filtered on 0.22-μm filters, inoculated with G. terrae, and incubated at 30°C for 48 h. Dilutions of the enriched extract were plated onto PYCa agar in soft agar containing G. terrae, and plaques were purified by five rounds of plaque assays (7). Periwinkle formed 3-mm turbid plaques on a lawn of G. terrae (7). Periwinkle has a *Siphoviridae* particle morphology, as determined by negative staining transmission electron microscopy. The particle has a 65-nm (standard error [SE], ±0.8 nm) icosahedral head and a 335-nm (SE, ±8.2 nm) flexible, noncontractile tail (*n* = 5).

A phenol-chloroform extraction method was used to extract DNA from a high-titer lysate before it was prepared for sequencing using the NEBNext Ultra II library preparation kit (New England BioLabs, Ipswich, MA) (8). Sequencing on an Illumina MiSeq platform yielded 168,288 single-end 150-bp reads. Newbler v2.9 and Consed v29 (9) were used for *de novo* assembly and checks for completeness, yielding a 55,657-bp genome with a GC content of 58.1%. Genome ends are defined by single-stranded 10-bp 3′ extensions (CTCGGGGCAT). Periwinkle shares >35% gene content with members of cluster DN in the Phamerator Actino_Draft database and was assigned to subcluster DN1 (4, 10, 11).

**FIG 1:**
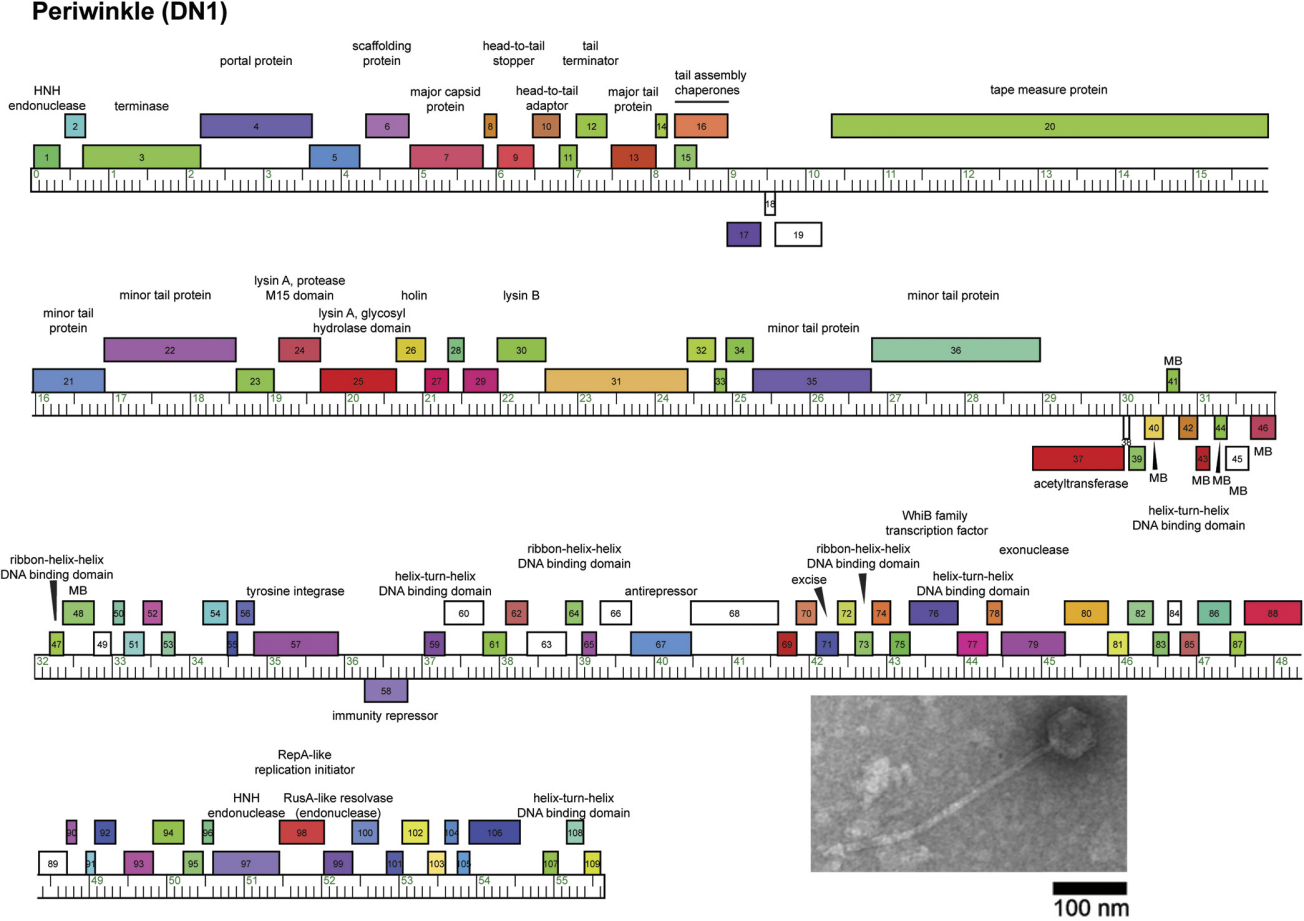
Genome map of *Gordonia* phage Periwinkle. The genome coordinates are represented by the ruler, in units of kilobase pairs. Forward and reverse genes are represented by colored boxes above and below the ruler, respectively. Genes were assigned to a phamily using Phamerator (10) with the Actino_Draft database, and different phamilies are indicated by different colors. Gene phamilies with only a single gene member (orphams) are represented by white boxes. Genes with transmembrane domains are labeled MB. An electron micrograph of Periwinkle is shown in the inset, with a scale bar of 100 nm.

The genome of Periwinkle was autoannotated using GLIMMER v3.02 and GeneMark v2.5 within DNA Master v5.23.6 (http://cobamide2.bio.pitt.edu) and PECAAN (https://blog.kbrinsgd.org/) before manual refining of translational starts based on inclusion of coding potential predicted by GeneMark.hmm and conservation across homologs according to BLAST and Starterator (http://phages.wustl.edu/starterator) (12–14). Putative gene functions were predicted using BLAST, TMHMM, and HHpred, and gene maps were prepared using the Phamerator database Actino_Draft (10, 15, 16). No tRNA genes were identified by ARAGORN v1.2.38 and tRNAscan-SE (17, 18). Periwinkle contains 109 protein-coding genes. The left arm of the genome contains mainly forward-transcribed assembly and structural genes (gp1 to gp36) (Fig. 1). All cluster DN phages contain at least one reverse-transcribed gene between the tail assembly chaperones and the tape measure protein, and Periwinkle contains three such genes (gp17 to gp19), including two orphams, i.e., gene phamilies with one member in the Actinobacteriophage Database (4, 11). The right arm contains forward-transcribed genes (gp59 to gp109), including five DNA-binding proteins (gp59, gp64, gp73, gp77, and gp108), an antirepressor (gp67), and a WhiB family transcription factor (gp76); gp57 and gp58 encode a tyrosine integrase and an immunity repressor, respectively, indicating that Periwinkle is likely a temperate phage (1).

The integrase and immunity repressor genes are located in an 8-kb region following the minor tail protein genes (gp35 and gp36) that contains forward- and reverse-transcribed genes (gp37 to gp58) that are likely expressed during lysogeny (19). Included are two DNA-binding proteins (gp42 and gp47), an acetyltransferase (gp37), and seven membrane proteins (gp40, gp41, gp44 to gp46, and gp48) that could contribute to superinfection immunity (19).

### Data availability.

Periwinkle is available in GenBank with the accession number ON456334 and the Sequence Read Archive (SRA) accession number SRR18715698.
